# Pain Perception During the Phases of Manual Reduction of Distal End Radius Fracture With a Periosteal Block

**DOI:** 10.7759/cureus.12691

**Published:** 2021-01-13

**Authors:** Wan Assanul Afzan Wan Ali, Elaine Zi Fan Soh, Shalimar Abdullah, Parminder Singh Gill Narin Singh, Amir Adham Ahmad, Jamari Sapuan

**Affiliations:** 1 Department of Orthopaedics and Traumatology, Universiti Kebangsaan Malaysia, Kuala Lumpur, MYS

**Keywords:** periosteal block, distal end radius fracture, pain perception, reduction

## Abstract

Introduction

Closed reduction is an effective method of treatment for distal end radius fractures. We present a case series of patients with distal end radius fractures who underwent closed manipulative reduction using the periosteal block. We describe the technique in detail and examine its efficacy in pain lowering effect during closed reduction.

Methods

Nineteen patients with distal end radial and ulnar fractures were included and grouped based on the Frykman classification. The reduction was performed using a periosteal block of 10 ml of 2% lignocaine injection. The severity of pain was recorded by utilizing the visual analog scale (VAS) in five phases: 1) before injection, 2) after 15 minutes of analgesia in a resting position, 3) during minimal motion, 4) during full manipulation and reduction, and 5) post-procedure. The VAS scoring was classified as painless (VAS score of 0), mild pain (VAS score between 1-3), and painful (VAS score of 4 and above).

Results

The study included 19 patients [median age of 53 years (range: 18-88 years)]; there were 11 (58%) males and eight (42%) females. The mechanism of injury was a fall (n=12, 63%) or a motor vehicle accident (n=7, 37%). There was a statistically significant reduction of pain between phase one and all the other phases. Between the different fracture configurations, there was no significant difference in pain reduction. The most painful phase was expected to be phase four, ie, during full manipulation, in which four (21%) patients had a VAS score of 0, 12 (63%) patients had a VAS score between 1-3, and three (16%) patients had a VAS score of 4. Thus, 16 out of 19 patients (84%) had no or minimal pain during the most painful phase. There were no complications from the periosteal blocks.

Conclusions

The periosteal nerve block is an effective procedure providing satisfactory analgesia during the reduction of distal radial and ulnar fractures. It has no side effects and is free from complications associated with conventional sedation.

## Introduction

One of the most common fractures seen in the emergency department is the distal radius and ulna fractures. It has a bimodal distribution with a higher incidence reported in children and the elderly [1,2]. In the elderly, the fracture is usually followed by a low-energy fall with an osteoporotic bone fracture rather than a high-energy trauma [2]. Frequently, closed manipulation and reduction are required for patients [3].

In many centers, sedation is the normal practice before the surgical procedure; however, it is associated with complications such as post-sedation nausea and vomiting and respiratory depression. Moreover, patients need to be monitored until they are fully conscious. As an alternative to sedation, the hematoma block is widely utilized due to its safety and simplicity [4,5]. However, it requires an actual hematoma formation where lignocaine is injected into the area of blood pooling, providing a very focused area of analgesia. Increasing the volume of analgesia could improve the analgesic effects although a pilot study on 20 patients showed no difference regarding the effect [6].

A new technique of periosteal block was devised by Tageldin et al. in 2015, where local anesthesia (lignocaine) was infiltrated around a greenstick fracture of the radius and ulna proximal to the fracture site [7]. There was no hematoma in this greenstick fracture precluding the use of a hematoma block. Closed reduction was performed without any additional analgesia or sedation. They reported excellent pain relief in 42 patients with distal end radius/ulna fractures without any complications or side effects. The potential complications of lignocaine are cardiac arrhythmias, hypotension, and central nervous system effects including tinnitus, dizziness, drowsiness, and convulsions [8].

There seems to be a wide variety of pain perception during fracture manipulation under hematoma block, with patients reporting highly varied mean visual analog scale (VAS) scores: 5.50 [6], 2.8 [9], 2.08 [5], and 0.97 [4]. Tageldin’s periosteal block reported a VAS score of 0 in 36 patients and a VAS score between 1-3 in six patients [7]. They did not measure the mean VAS score. Another procedure similar to the periosteal block is the wide-awake local anaesthesia no tourniquet (WALANT) technique utilized for bony surgery. WALANT is a technique popularised by Donald Lalonde, and it involves adding adrenaline to lignocaine [10]. Adrenaline causes a vasoconstrictive effect to allow for a relatively bloodless field for surgery. However, it is mostly applied in soft tissue and tendon surgery rather than bony surgery.

A few studies have reported the use of WALANT in distal radius plating. A single patient in a case report by Ahmad et al. [11] reported a VAS score of 0 while Huang et al. reported 24 cases but only assessed postoperative pain score on day one post-surgery and not during the surgery [12].

The objective of this study was to evaluate the efficacy of using the periosteal block in the reduction of distal end radius fractures. Specifically, we wanted to assess pain scores at different phases of the fracture manipulation and in relation to fracture patterns.

## Materials and methods

The study included patients aged 18 years and above with closed displaced distal radial fractures (with or without associated distal ulnar fractures) that required a closed reduction either as a temporary or definitive treatment. The exclusion criteria were as follows: patients with multiple fractures, those with ipsilateral shaft fracture, patients requiring repeat CMR, unconscious patients, open fractures, undisplaced fractures not requiring CMR, patients who refused local anesthesia, and patients with clinical evidence of compartment syndrome and allergy to local anesthesia. This study was approved by our university's ethical committee (ethical code: PPI/111/8/JEP-2017-284).

The case series consisted of 19 consecutive patients over a period of six months. Fractures were classified according to the Frykman classification (Table 1). All 19 patients underwent periosteal blocks using the described technique followed by fracture immobilization either using a slab or cast. Informed consent was obtained from all the patients.

Patients were required to grade their levels of pain using VAS (0-10, ranging from no pain to severe pain). Pain scores were recorded in five different procedural phases: 1) before the periosteal injection, 2) after 15 minutes of analgesia infiltration in a resting position, 3) during minimal motion and manipulation, 4) during full manipulation and reduction of the fracture fragments, and 5) post-procedure. Patients were briefed about the procedure in advance and they understood the different phases where they would be asked about the VAS score. The VAS scoring was then classified as painless (VAS score of 0), mild pain (VAS score between 1-3), and painful (VAS score of 4 and above). If the patient experienced a painful procedure (VAS score of 4 or above), intravenous ketorolac 30 mg stat was given as a rescue dose. Ketorolac was selected as the drug of choice as it has no side effects of nausea and vomiting, which are possible side effects of lignocaine toxicity. All periosteal blocks were performed by a single operator with at least five years of experience in orthopaedics and trained to perform periosteal blocks. Vital signs and any side effects and toxicity symptoms of local anaesthesia were monitored, including cardiotoxicity, central nervous toxicity, and allergic reaction. The procedure was performed in the emergency department with cardiac monitoring and an emergency trolley with lipid emulsion available as an antidote [13].

Periosteal block technique (adapted from Tageldin et al. [7])

**Figure 1:**
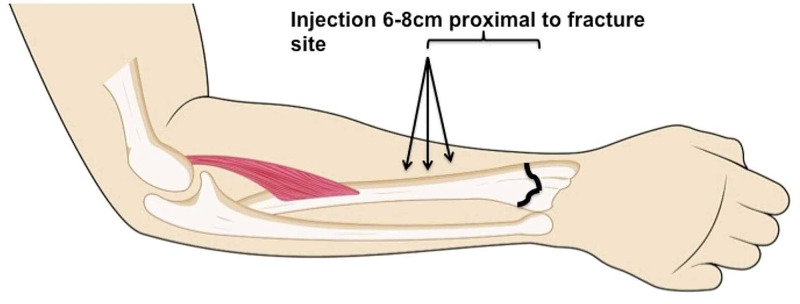
Schematic diagram depicting the site of injection 6-8 cm proximal to the fracture site An injection of 2% pure lignocaine was given straight perpendicularly (3 ml), angled volarly (3.5 ml), and angled dorsally (3.5 ml). The total volume of lignocaine injected was 10 ml

The patient was placed in a supine position with the injured hand and forearm resting on a dressing table. A precautionary intravenous cannula was inserted into the uninjured arm. Oxygen and intravenous fluids were available. Local anesthesia was infiltrated based on the universal standard aseptic technique with 2% lidocaine at a dosage of 2 mg/kg of body weight in a volume of 10 ml for a single bone [6,7,14]. The injection site was made between 6-8 cm proximal to the fracture site (Figure 1). The needle was inserted from the lateral aspect perpendicular to the radius straight down until it touched the periosteum of the radius. Approximately 3 ml of lidocaine was injected. The needle was then removed and re-inserted 1 cm dorsally and advanced dorsally while infiltrating about 3.5 ml of lidocaine. This step was then repeated 1 cm volar to the original site and the needle advanced while infiltrating 3.5 ml of lidocaine volarly. The injection sites near-circumferentially covered the distal third shaft of the radius at the lateral, volar, and dorsal regions. If the ulna styloid was fractured, an additional 5 ml of lignocaine was injected. There was little risk of injuring the radial and ulnar arteries and nerves as the injection was performed circumferentially around the radius and ulna bone.

We then waited 15 minutes before minimally mobilizing the forearm, which was followed by close manipulation and reduction. After the reduction, casting or a splint was applied. The patient was observed in an observation room for two hours before being discharged. Any complications or side effects were recorded.

All statistical analyses were performed with SPSS Statistics 23.0 for Windows (IBM, Armonk, NY) and Microsoft Excel 2003 (Microsoft Corporation, Redmond, WA). The Wilcoxon signed-rank test and the Kruskal-Wallis test were used to analyze data.

## Results

A total of 19 patients were included in the study. The median age of the patients was 53 years (range: 18-88 years). Seven (37%) patients were below 50 years old and 12 (63%) patients were above 50 years old. There were 11 (58%) males and eight (42%) females. With regard to the mechanism of injury, 12 (63%) patients sustained the fractures following a fall and seven (37%) following motor vehicle accidents. The demographic data are presented in Table 1.

All patients were further subdivided into eight groups based on their fracture configuration (Frykman classification). Group II (extra-articular distal end radius fracture with ulna fracture) had the highest number of patients (seven, 36%). There were no patients in groups I, VII, and VIII (Table 1).

**Table 1:** Demographic data depicting age, gender, Frykman classification, and the mechanism of injury MVA: motor vehicle accident

Variables		Frequency	Percentage (%)
Age	18–50 years	7	36.8
	>50 years	12	63.2
Gender	Male	11	57.9
	Female	8	42.1
Frykmann classification	1	0	0
	2	7	36.8
	3	3	15.8
	4	4	21.1
	5	1	5.3
	6	4	21.1
	7	0	0
	8	0	0
Mechanism of injury	Fall	12	63.2
	MVA	7	36.8
Total		19	100.0

**Figure 2:**
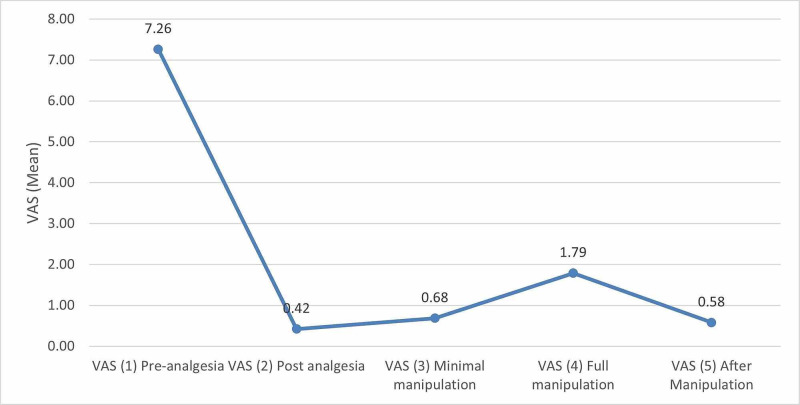
Graph depicting the comparison of pain scores (VAS) in all patients VAS: visual analog scale

**Figure 3:**
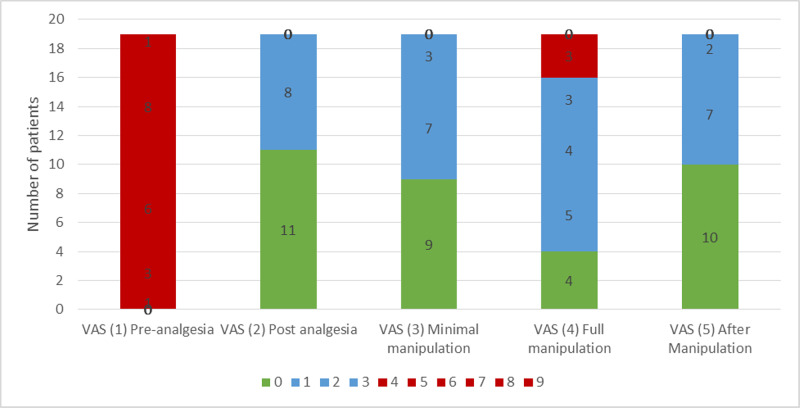
Bar chart depicting pain severity in each of the reduction phases VAS: visual analog scale

There was significant pain reduction seen in each of the phases (Figure 2). A paired t-test between phases two to five compared to phase one (basic pain pre-analgesia) was performed (Table 2). All groups showed significant pain reduction in all phases (Figure 3). There was no significant difference in pain reduction between fracture configurations. Table 3 shows pain scores in patients grouped by fracture configuration. The t-test was used to compare phases two to five with phase one.

**Table 2:** Paired t-test comparing pain scores (VAS) between the phases *Statistically significant VAS: visual analog scale

	N	Mean	Standard deviation	T	P-value
VAS (1): pre-analgesia	19	7.26	0.99		
VAS (2): post-analgesia	19	0.42	0.51	22.90	0.001*
VAS (3): minimal manipulation	19	0.68	0.75	19.57	0.001*
VAS (4): full manipulation	19	1.79	1.40	12.21	0.001*
VAS (5): after manipulation	19	0.58	0.69	20.02	0.001*

**Table 3:** Comparison of pain scores between the phases in each of the fracture configurations *Statistically significant VAS: visual analog scale

Frykman classification		N	Mean	Standard deviation	T	P-value
2	VAS (1): pre-analgesia	7	6.57	1.13		
	VAS (2): post-analgesia	7	0.43	0.53	10.328	0.001*
	VAS (3): minimal manipulation	7	0.71	0.95	8.312	0.001*
	VAS (4): full manipulation	7	1.57	1.81	5.123	0.002*
	VAS (5): after manipulation	7	0.57	0.79	9.165	0.001*
3	VAS (1): pre-analgesia	3	7.67	0.58		
	VAS (2): post-analgesia	3	0.00	0.00	23.000	0.002*
	VAS (3): minimal manipulation	3	0.00	0.00	23.000	0.002*
	VAS (4): full manipulation	3	0.00	0.00	23.000	0.002*
	VAS (5): after manipulation	3	0.00	0.00	23.000	0.002*
4	VAS (1): pre-analgesia	4	8.00	0.82		
	VAS (2): post-analgesia	4	0.50	0.58	11.619	0.001*
	VAS (3): minimal manipulation	4	1.00	0.82	9.899	0.002*
	VAS (4): full manipulation	4	2.50	1.29	5.284	0.013*
	VAS (5): after manipulation	4	0.75	0.96	8.490	0.003*
6	VAS (1): pre-analgesia	4	7.50	0.58		
	VAS (2): post-analgesia	4	0.50	0.58	17.146	0.001*
	VAS (3): minimal manipulation	4	0.75	0.50	14.100	0.001*
	VAS (4): full manipulation	4	2.00	0.82	8.521	0.003*
	VAS (5): after manipulation	4	0.75	0.50	14.100	0.001*

**Figure 4:**
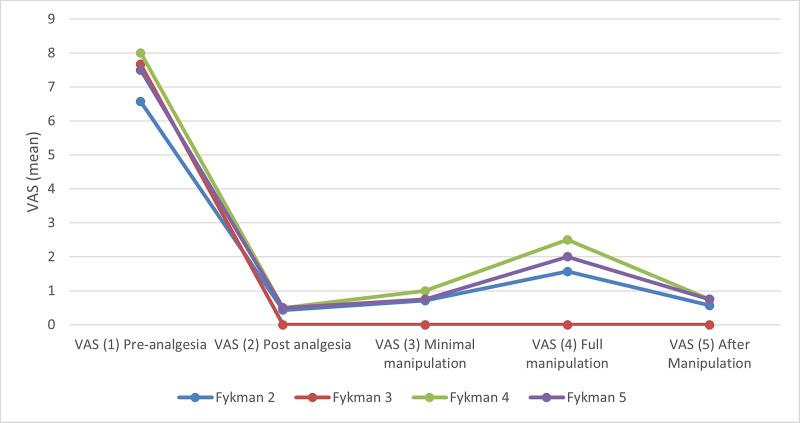
Comparison of the pain score progression in each of the fracture configurations VAS: visual analog scale

With regard to pain experienced in all the five phases, the VAS score was grouped as painless (VAS score of 0), mild pain (VAS score between 1-3), and painful (VAS score of 4 and above) (Figure 4). In phase two (post-analgesia), phase three (minimal manipulation), and phase five (after manipulation), none of the patients experienced a VAS score of 4 and above, which was categorized as painful. The most painful phase was expected to be phase four, ie, during full close manipulation and reduction. In this phase, four (21%) patients had a VAS score of 0, 12 (63%) patients had a VAS score between 1-3 and three (16%) patients had a VAS score of 4. Thus, 16 out of 19 patients (84%) had no or minimal pain during the most painful phase. There were no complications from the periosteal blocks.

## Discussion

Most of the distal radius and ulna fractures can be treated in an outpatient setting although 20% of these cases have to be admitted, ie, open fractures, complex fracture configurations requiring surgery, associated polytrauma, or patients with multiple comorbidities for observation post-fracture reduction [15]. With adequate analgesia, fractures can be reduced or a cast or splint applied for temporary or definitive treatment.

Multiple methods of analgesia are available, which can be classified as follows: 1) procedural sedation (using a combination of opioids and benzodiazepine) and 2) neural blocks, which are further grouped into i) regional (Bier, axillary, supraclavicular/brachial plexus) and ii) local block (hematoma block). Hematoma blocks offer a better quality of reduction and similar effectiveness for pain relief compared to brachial plexus block with the advantage of having no motor paralysis [5,16]. Hematoma blocks are better than Entonox, with significantly less pain and better patient acceptance [6]. Bier’s block is associated with a shorter length of stay in the emergency department compared to ketamine [17]. A study by Mohr on 1,804 patients undergoing Bier’s block reported only nine patients with adverse effects, and the procedure was considered safe and effective [18].

Procedural sedation remains the most popular method of analgesia [19]. Although procedural sedation has previously been shown to provide excellent pain relief, it can lead to several side effects, such as nausea and vomiting, respiratory depression, dysphoria, and hallucinations. Furthermore, using procedural sedation also consumes emergency department resources and time as patients have to be observed for quite a long period before they can be safely discharged home [10,19-20]. None of the techniques mentioned above (hematoma block, intravenous regional anaesthesia, regional nerve blocks, sedation, and general anaesthesia) has any sort of superiority over the other [19].

Similar to the findings of Tageldin’s study, we too found that the proximal periosteal block is a very acceptable method of facilitating distal end radius fracture reduction. It provides an excellent mode of pain relief, resulting in high patient satisfaction levels. All patients do not require anxiolytic or sedation such as benzodiazepine during closed reduction. This will reduce complications related to drugs, especially opioids. It also facilitates post-reduction treatment as there is no need for post-sedation observation. In our center, administering sedation requires the patient to be transferred from the green zone to the yellow zone where the patient is then attached to a blood pressure cuff and pulse oximeter with an emergency trolley on standby. Therefore, apart from providing excellent pain reduction, the periosteal block also significantly shortens the total treatment duration in the emergency department.

There seems to be a wide variety of pain perception during fracture manipulation under a hematoma block. A pilot study by Orbach et al. with a hematoma block reported a mean VAS score of 5.50 in one group (10 mL of 2% lidocaine) and 3.09 in another group (20 mL of 1% lidocaine) although the difference was not statistically significant [6]. Another study revealed an average VAS score of 7.19 for the Entonox group and 2.8 for the hematoma block group [9]. A study comparing hematoma block and brachial plexus block reported a mean VAS score of 2.08 for the hematoma block and 1.60 for the brachial plexus block [5]. The lowest VAS score was seen in a study by Myderizzi et al. (VAS score of 0.97 during fracture manipulation) [4].

Tageldin et al.’s periosteal block reported a VAS score of 0 in 36 patients and a VAS score between 1-3 in six patients [7]. None had a painful VAS, which was categorized as 4 and above. However, during phase four of manipulation in our study, we had three patients (15.8%) with a VAS score of 4. These three patients required intravenous ketorolac rescue analgesia and completed the reduction without sedation.

Additionally, we observed that pain was associated with extensive soft tissue injury. In the three patients mentioned above, the fracture sites were extensively swollen with multiple bruises compared to other patients. This might indicate that the periosteal block may effectively block pain stimulus caused by bone fractures and joint capsule rather than the surrounding soft tissue. We did not record the degree of soft tissue injury in this study.

Our study showed a statistically significant decrease in pain scores with the periosteal block in all the four points phases: 15 minutes post-analgesia, post-analgesia with minimal manipulation, during manipulation, and 15 minutes after manipulation. The results of our study are comparable with that of Tageldin et al., and we have taken a step further by performing a statistical analysis.

In the study by Bajracharya et al., one out of 50 patients had an infection after a hematoma block, requiring drainage and external fixation [5]. Thus, there was some concern that our periosteal block could potentially convert a close fracture into an open fracture with the infiltration of lignocaine. However, the infiltration was made 6-8 cm proximal to the fracture site and outside the reactive zone. Therefore, there was a very low risk of causing an infected hematoma surrounding the fracture site. To date, there is no comparable study related to post-periosteal block infection. We used the universal standard aseptic technique for administering the periosteal block.

All of the blocks were performed by the main author. However, team members who observed the procedure felt confident of repeating the technique although this was not assessed. We did not assess anxiety levels, which would have been interesting as Celik et al. have reported higher anxiety levels in females and those with higher education [15]. Davison et al. have reported that patients who underwent sedation compared to local anaesthesia had higher preoperative anxiety levels [10]. Limitations of this study include the small number of patients and a lack of comparison with the standard technique of procedural sedation or a hematoma block; another limitation was that the same dosage of the drug was used irrespective of the type and severity of fractures.

## Conclusions

The periosteal nerve block is an effective method for providing excellent pain relief during the phases of manual reduction of distal radial and ulnar fractures. It can be used in all types of fracture configurations in the Frykman classification and provides similar outcomes. However, we have not compared periosteal blocks with any other modality of treatment.

The periosteal nerve block provides an alternative to the conventional sedation method and can avoid the complications associated with conventional sedation. Even though this study was performed by orthopaedic doctors, we believe that with proper training and education, the periosteal nerve block can be performed by any medical personnel in an emergency setting.
